# The challenge of studying complex diseases undergoing complex treatments: the metastatic cancer model

**DOI:** 10.1186/1479-5876-10-S2-A46

**Published:** 2012-10-17

**Authors:** Ena Wang, Francesco M Marincola

**Affiliations:** 1 Infectious Disease and Immunogenetics Section (IDIS), Department of Transfusion Medicine, Clinical Center and trans-NIH Center for Human Immunology (CHI), National Institutes of Health, Bethesda, Maryland, USA

## 

A cancer immune signature implicating good prognosis and responsiveness to immunotherapy was described that is observed also in other aspects of immune-mediated, tissue-specific destruction (TSD) [1,2]. Its determinism remains, however, elusive. On one side it appears that the genetic background of the host’s bears significantly on immune responsiveness, on the other it appears that tumor can behave differently within the same genetic background (as in the case of mixed responses). This apparent paradox can only be explained by a multi-factorial model of cancer immune responsiveness [3]. It should be emphasized that host and cancer genetics are largely overlapping since cancer cells carry the majority of the host’s genetics. Thus, inherited genetic factors may affect the biology of cancer cells besides that of normal cells. It could be postulated that some patients carry a genetic background that make them resistant to immunotherapy by effecting either the biology of the immune response, the biology of the cancer cells or both. On the other hand, “an immune-responsive genotype” may still be limited by the genetics of the tumors: in other words, although the patient may be predisposed to cancer rejection the tumor lacks additional properties necessary for its recognition by the immune response [4]. In this model, a favorable genetic background of the host is necessary but not sufficient for tumor rejection as the possession of a shotgun is necessary to shoot a duck but at the same time a skill in shooting is required. A good example is provided by the analysis of patients with IRF-5 polymorphism [5]; the “immune resistant phenotype” appears to almost exclusively preclude cancer rejection during adoptive therapy with tumor infiltrating lymphocytes; however, “the immune responsive phenotype” can be segregated into two categories; one enriched in patients responding to therapy and the other of non-responding [6,7]. Although, other host’s genetic factors could be responsible for this sub-classification, it is also possible that, given a favorable genetic background, the genetics of the tumor may become the determining factor [8].

We recognize that this classification of factors that may influence immune responsiveness may be too rigid. In reality, immune responsiveness may depend upon a continuum determined by the interaction of a multitude of factors that for simplicity can be separated into broad categories depending upon the host’s genetic background, somatic mutations, and external factors such as intensity and effectiveness of treatment, general condition of the patient and a multitude of other hidden co-factors. In the presentation at the NY Academy of Sciences we will present our strategy to dissect the question of cancer immune responsiveness by study dynamically the behavior of human cancers under natural conditions on in response to therapy.

## References

[B1] Wang E Worschech A Marincola FM The immunologic constant of rejection Trends Immunol 2008 29 256 262 10.1016/j.it.2008.03.002 18457994

[B2] Immunologic signatures of rejection 2010 Springer. New York, NY

[B3] Wang E Marincola FM Bottom up: a modular view of immunology Immunity 2008 29 9 11 10.1016/j.immuni.2008.07.002 18631452 PMC3417224

[B4] Wang E Uccellini L Marincola FM A genetic inference on cancer immune responsiveness Oncoimmunology 2012 in press 10.4161/onci.19531 PMC3382909 22754772

[B5] Uccellini L Erdenebileg N De Giorgi V Tomei S Ascierto ML Bedognetti D Liu Q Wang E Marincola FM Rosenberg SA IRF-5 gene polymorphism and melanoma 2011 in preparation

[B6] Wang E Marincola FM A natural history of melanoma: serial gene expression analysis Immunol Today 2000 21 619 623 10.1016/S0167-5699(00)01724-2 11114422

[B7] Wang E Miller LD Ohnmacht GA Mocellin S Petersen D Zhao Y Simon R Powell JI Asaki E Alexander HR Prospective molecular profiling of subcutaneous melanoma metastases suggests classifiers of immune responsiveness Cancer Res 2002 62 3581 3586 12097256 PMC2241738

[B8] Ascierto ML De Giorgi V Liu Q Bedognetti D Murtas D Chouchane L Wang E Marincola FM An immunologic portrait of cancer J Transl Med 2011 9 146 10.1186/1479-5876-9-146 21875439 PMC3175185

